# From gesture to meaning: art therapy, children’s drawing process, and emotional resilience—a phenomenologically informed psychological study

**DOI:** 10.3389/fpsyg.2026.1940689

**Published:** 2026-10-15

**Authors:** Wenke Sun

**Affiliations:** Department of Fine Art and Design, Wenzhou University, Wenzhou, Zhejiang, China

**Keywords:** art therapy, children’s drawings, emotional resilience, meaning-making, phenomenology, psychological well-being

## Abstract

Children often struggle to verbalise difficult emotions, which makes therapeutic methods that support their emotional health and wellbeing particularly important. This study examined the relationship between art therapy, children’s drawing processes, and emotional resilience from a phenomenologically oriented psychological perspective in China. The study aimed to understand children’s experiences of drawing during art therapy and the role of these experiences in meaning-making and the development of resilience. A qualitative phenomenological design was used with 20 children (aged 8–12 years) who took part in a 10-week art therapy programme delivered across three settings. Data were collected through participant observation, semi-structured interviews, reflective dialogue, and analysis of 200 drawings, and were analysed following transcendental phenomenological procedure. Four interconnected themes emerged: Drawing as Emotional Externalisation, Symbolic Meaning-Making Through Visual Narratives, Transformation of Fear into Agency, and Emergence of Emotional Resilience and Future Orientation. Participants described drawing as a means of externalising difficult emotions, constructing personal meaning through symbols and stories, and developing a stronger sense of agency and coping. The findings suggest that art therapy can function as a developmental bridge connecting emotion and meaning, supporting children’s experienced sense of resilience through emotional awareness, reflective understanding, and self-expression. As a qualitative study without a comparison group or independent outcome measures, the findings describe children’s perceived and experienced changes rather than demonstrated therapeutic effects; with this caveat, the results carry exploratory implications for education, counselling, and therapeutic practice aimed at supporting children’s psychological wellbeing.

## 1 Introduction

Emotions, interpersonal experiences, and self-perceptions are intertwined with children’s developing psychological identities (Cillessen and Bellmore, 2004). Self-perception, expression, and regulation of emotions during these early years have a profound impact on later mental health, social adaptation, and functioning (Aghaziarati and Nejatifar, 2023; Thümmler et al., 2022). However, children’s emotional experiences have not always been easy to express and communicate verbally (Sravanti, 2026; Abidi, 2025). Psychological research has therefore increasingly focused on alternative means of expression that allow children to communicate their experiences (Volkmer et al., 2024). Art therapy stands out as a distinctive approach, as it treats the creative expression of art not merely as an interest but as a valuable process for externalising, exploring, and transforming internal experiences (Borovica et al., 2026). Arts-based interventions increasingly demonstrate therapeutic benefits for promoting emotional expression, self-awareness, and psychological wellbeing in children and adolescents (Javadian et al., 2025). Creativity has been shown to help children express feelings, understand themselves, and reflectively construct meaning, all of which support social adjustment (Sternfeld et al., 2024). Drawing is a particularly important expressive medium and a form of communication that differs from verbal communication by enabling children to represent experiences symbolically, visually, and metaphorically (Terton et al., 2022; Trifunović et al., 2022). In recent years, children’s drawings have increasingly been recognised as dynamic processes of meaning-making rather than static products (Valquaresma, 2024). Art-based interventions are attracting growing international interest, mirroring a wider focus on children’s emotional needs amid significant social change (Moula et al., 2022).

Although the value of art therapy is increasingly recognised, gaps remain in the literature. Most empirical studies of art therapy with children have focused on objective outcomes, including reductions in behavioural problems, increases in emotional expression, or changes in coping strategies (Brechet et al., 2022; Cortés and Pérez, 2025; Mastrothanasis et al., 2025a, 2025b). Quantitative indicators can demonstrate that change has occurred, but they can fail to capture the processes by which drawing becomes psychologically meaningful, or the mechanisms through which resilience develops during creative engagement (Brechet et al., 2022; Sheridan and Van Lith, 2025). Past studies have also tended to emphasise therapist interpretations, clinical evaluations, and programme effectiveness, with comparatively less research exploring children’s own perspectives on the role of drawing in emotional understanding and change (Razali et al., 2025; Sahai and Tiwari, 2026). This gap is more pronounced in Asian contexts such as China, where phenomenological studies of children’s lived experiences in art therapy remain relatively limited.

A further limitation concerns the tendency to conceptualise emotional expression as distinct from the process of making sense of emotion. Research often emphasises art therapy’s capacity to release emotion, while reporting comparatively less on how emotions are woven into coherent stories that support resilience (Razali et al., 2025; Brechet et al., 2022). Emotional expression alone does not guarantee psychological growth. Drawing can serve as a connecting thread between the emotional world and reflective thinking, allowing children not only to express emotions but also to form new relationships with them (Sheridan and Van Lith, 2025; Cao and Navarro, 2025; Sahai and Tiwari, 2026). Capturing this process requires methodologies able to attend to subjective experience, symbolic communication, and shifting personal interpretation. Phenomenology is particularly well suited to this purpose, as it seeks to understand phenomena as they are experienced by the people living them, rather than from the perspective of an outside observer (Mitchell and Meehan, 2022; Pipere et al., 2025).

Building on a psychological and phenomenological understanding of children’s drawing processes, the present study aims to address these gaps by examining the relationship between art therapy, children’s drawing processes, and emotional resilience from a Chinese perspective. Rather than treating drawings as diagnostic indicators or objective measures, the study focuses on children’s experiences of drawing, the emergence of meaning within drawing, and the contribution of these experiences to the development of resilience. The study proceeds from the premise that resilience is not solely the result of external intervention but arises from children’s active engagement in processes of expression, reflection, and interpretation. Understanding these processes may offer useful insight for art therapists, educators, school counsellors, and mental health practitioners seeking developmentally sensitive ways of supporting children’s wellbeing.

The theoretical grounding for this study draws on phenomenology (Moustakas, 1994), expressive arts therapy (Malchiodi, 2003), and resilience theory (Rutter, 1985). The phenomenological approach involves studying lived experience and the meanings people attach to phenomena encountered in their everyday world (Finlay, 2009). From a phenomenological perspective, drawings can be understood as “expressions of experience” that help define how children encounter, interpret, and understand their emotional world (Darling-McQuistan, 2017). Expressive arts therapy offers a complementary lens, focusing on the process of art-making as a means of revealing thoughts, feelings, memories, and relationships that can be explored and transformed (Estrella, 2023); in this view, art functions as a communication tool supporting self-realisation and psychological integration. Resilience theory contributes a further perspective, positing that the capacity to cope with stress, to make meaning, and to draw on supportive relationships are all aspects of an ongoing, dynamic process of adaptation (Theron and Theron, 2014). Together, these perspectives suggest that drawing can act as a mediating process through which emotional experiences are reflected upon, interpreted, and integrated into a more resilient sense of self.

The following research questions guided the study: (1) What are children’s experiences of drawing during art therapy sessions? (2) What meanings do children assign to the symbols, stories, and images that appear in their drawings? (3) How are emotions expressed, examined, and transformed through the act of drawing? (4) How does art therapy contribute to the development of children’s emotional resilience? (5) How do these experiential mechanisms relate to artistic expression, meaning-making, and resilience development? To address these questions, the study pursued five objectives: to understand children’s lived experiences of drawing during art therapy; to examine the symbols and narratives present in children’s artwork; to investigate how meaning is constructed through this artwork; to understand the role of art therapy in emotional resilience; and to develop a phenomenologically informed account of the relationship between drawing, meaning-making, and resilience among children in China.

## 2 Literature review

Recent research consistently shows that art-based interventions support emotional expression, meaning-making, and resilience across diverse populations and settings, although researchers have approached these outcomes from different theoretical perspectives and research designs, producing complementary but distinct accounts of how art supports psychological adaptation. Emotional release is a recurring theme in the literature, alongside a parallel process through which people reconstruct meaning, develop self-awareness, and build adaptive capacity. Quantitative studies have generally focused on observable outcomes and changes in emotional regulation or resilience, while qualitative and phenomenological studies have offered more in-depth insight into the subjective experiences underlying such changes.

Metaphor and symbolic representation have been highlighted across several studies as mechanisms for building resilience. Sheridan and Van Lith (2025) examined the therapeutic influence of metaphor in an online painting intervention with university students who had experienced highly stressful or traumatic events. Quantitative results showed no significant improvement in resilience scores, but qualitative findings indicated gains in affect recognition, acceptance of circumstances, and the reframing of life experiences. Similarly, Cao and Navarro (2025) studied metaphor within the Brundibár Project, involving 217 children and adolescents who had experienced trauma, and found that metaphor served as a powerful tool for narrative reconstruction, symbolic reframing of difficult experiences, and the creation of alternative interpretations. In both studies, resilience appeared to emerge not solely from emotional disclosure but from symbolic transformation and reinterpretation; Cao and Navarro emphasised the collective and developmental aspects of metaphor construction in an extended intervention with vulnerable young people, while Sheridan and Van Lith focused on individual psychological change within a shorter intervention.

The nonverbal nature of drawing has also been emphasised in research on emotion regulation. Razali et al. (2025) found that structured and semi-structured drawing activities allowed participants to access repressed memories and emotions within a supportive space, reducing the pressure of direct verbal disclosure and leading to improved emotional regulation, coping, and self-esteem. Sahai and Tiwari (2026) reported comparable findings in a case study of an adolescent in residential treatment, in which symbolic artistic expression supported exploration of emotion, self-reflection, and the development of a more coherent sense of self. Across both studies, drawing was positioned as a medium connecting internal emotional experience with conscious awareness; while Sahai and Tiwari focused on an individual case, Razali et al. examined collective workshop experiences and trauma processing. Taken together, these studies suggest that drawing functions as an expressive and reflective activity supporting psychological development across a range of settings and populations.

Experimental research has provided further evidence of drawing’s role in emotional regulation and coping. Brechet et al. (2022) examined drawing as an emotion regulation strategy in children and found that it effectively reduced negative mood following mood induction; distraction-oriented drawing tasks produced greater emotional benefit than venting-oriented tasks, particularly among children with moderate emotional comprehension. These findings indicate that drawing can regulate emotion beyond simple expression. While clinical and qualitative research has tended to emphasise meaning-making and symbolic exploration, Brechet et al.’s work demonstrates that drawing can also have measurable regulatory effects on emotional states. Comparable structured interventions have strengthened emotion regulation in school-aged children (Sepanta et al., 2020) and reduced separation anxiety through attachment-based play therapy built on nonverbal, expressive activities (Abdolali et al., 2023).

Phenomenological investigations have further illuminated the experiential dimensions of artistic engagement. In a study of mental health service users’ experiences in studio-based art groups, Mitchell and Meehan (2022) found that art-making generated a sense of meaning, belonging, and new possibilities for self-understanding, with participants describing art-making as supportive of both pre-reflective and reflective aspects of identity. Similarly, Pipere et al. (2025) used interpretative phenomenological analysis to examine arts therapists’ experiences of working with Ukrainian war refugees, highlighting self-reflection, perceived value to clients, and the potential of creative interventions during trauma and displacement. Although conducted with different populations, these studies demonstrate that artistic processes can support meaning-making, identity reconstruction, and adaptation in difficult circumstances, and that the experiences of those engaged in the artistic process are themselves of central interest, not merely programme outcomes.

The broader literature on participatory arts similarly suggests that creative engagement fosters resilience through social, emotional, and educational pathways. Drama interventions have been shown to enhance communication, adaptability, emotional expression, and collective resilience in school communities during periods of disruption (Mastrothanasis et al., 2025a), while puppetry has supported students with complex educational needs in self-expression, social participation, emotional support, and behavioural adjustment (Mastrothanasis et al., 2025b). Cortés and Pérez (2025) found that colour-based journaling was associated with increased emotional awareness, coping, communication, and perceived resilience among vulnerable children in foster care over the course of a year, while Aydos and Özet (2025) found that children’s colour choices and drawings reflected highly personalised emotional constructions grounded in their experiences and relationships. Collectively, these studies indicate that art can support resilience in multiple ways — as a form of emotional expression, a means of self-understanding, a vehicle for social connection, and a tool for adaptive meaning-making. However, comparatively little research has examined children’s own experiential accounts of drawing as a process of moving from emotional expression toward a lived sense of resilience, particularly within specific cultural contexts such as China. Phenomenologically grounded studies of how lived meaning is constructed through children’s drawing experiences, and how artistic engagement supports emotional resilience within particular cultural settings, therefore remain needed. Engagement with painting and other art forms has likewise been shown to strengthen the link between purpose in life and hope among young people (Ercole and Pranoto, 2025).

## 3 Methodology

This study used a qualitative phenomenological research design to examine children’s experiences and processes within art therapy and the contribution of these experiences to the development of emotional resilience. Phenomenology was selected because the primary aim of the research was to understand the lived experiences, meanings, perceptions, and interpretations that children attach to artistic expression, rather than to measure predetermined variables.

The study was conducted across three therapeutic, child-oriented settings in eastern China: two school-based counselling centres and one community-based art therapy centre. These settings were selected because they offered structured art therapy programmes designed to support children’s emotional development and psychological wellbeing. Children aged 8–12 years were the focus of the study (Table 1). Inclusion criteria required at least 8 weeks of participation in art therapy sessions, the ability to take part in an interview, and the provision of parental consent and child assent. Children experiencing acute psychiatric crises, and those with severe cognitive impairments that could affect participation, were excluded.

**Table 1:** Participant profile.

Participant code	Age	Gender	Number of drawings	Interview duration (minutes)
P1	8	Female	10	28
P2	9	Male	10	31
P3	10	Female	10	35
P4	11	Male	10	33
P5	8	Female	10	26
P6	12	Male	10	38
P7	10	Female	10	32
P8	9	Male	10	29
P9	11	Female	10	34
P10	12	Male	10	40
P11	8	Female	10	27
P12	10	Male	10	31
P13	11	Female	10	36
P14	9	Male	10	30
P15	12	Female	10	41
P16	8	Male	10	25
P17	10	Female	10	32
P18	11	Male	10	34
P19	9	Female	10	29
P20	12	Female	10	39

Purposive sampling was used to recruit information-rich participants with first-hand experience of the phenomenon under study. Twenty children were recruited in partnership with therapists and school counsellors, who screened for eligibility using the inclusion criteria described in Table 2.

**Table 2:** Emergent themes.

Theme	Subthemes/codings
Drawing as emotional externalisation	Releasing hidden feelings; communicating without words; safe emotional expression
Symbolic meaning-making through visual narratives	Personal symbols; story construction; reinterpreting experience
Transformation of fear into agency	Confronting difficult emotions; regaining control; developing courage
Emergence of emotional resilience and future orientation	Positive self-identity; hopefulness; adaptive coping

### 3.1 Researcher role, positionality, and reflexivity

The first author planned and coordinated the study and holds training in both art-based therapeutic facilitation and qualitative research methods, but did not act as the children’s primary therapist during the programme. Art therapy sessions were delivered by qualified art therapists and counsellors employed at each of the three settings, following a shared session structure and thematic protocol developed for this study. The researcher was present during sessions as a participant-observer, recording field notes on interaction, engagement, and emotional expression, and independently conducted the semi-structured interviews and the subsequent analysis of interview transcripts, field notes, reflective dialogue, and drawings once each ten-week programme had concluded. Separating the delivery of therapy from the conduct of research in this way was intended to reduce the likelihood that the researcher’s dual interest as both facilitator and evaluator would shape participants’ responses.

Because phenomenological and visual analysis are inherently interpretive, the researcher’s professional background in art education and expectations regarding the therapeutic value of drawing could plausibly have influenced data collection and interpretation. Several reflexive strategies were used to manage this influence. Before formal analysis began, the researcher engaged in epoché (bracketing) by keeping a reflexive journal in which personal assumptions, prior professional experience, and expectations about likely findings were made explicit and set aside as far as possible at each stage of coding. During analysis, a second qualitative researcher independently reviewed a subset of transcripts and drawings and compared codes with the first author; disagreements were resolved through discussion and, where necessary, by returning to the original data. Member checking was carried out with children, and with parents where appropriate, by sharing brief plain-language summaries of emerging interpretations and inviting correction or elaboration. These procedures do not remove the interpretive role of the researcher, but were intended to increase the transparency and trustworthiness of the analysis.

The art therapy intervention ran for 10 consecutive weeks, with each session lasting approximately 60–90 min. The programme comprised developmentally appropriate drawing activities designed to encourage emotional awareness, symbolic expression, reflection, and resilience-building. Session themes included “My World,” “Draw Your Feelings,” “Safe Place Drawing,” “Family Story Drawing,” “Fear and Courage,” “Challenge and Strength,” “Future Self,” “Journey Drawing,” “Resilience Tree,” and “Who Am I Now?” These activities enabled children to express feelings, relationships, fears, dreams, and coping strategies through imagery, with the therapeutic process emphasising self-expression and the use of personal symbols, narratives, and imagery for meaning-making rather than artistic skill.

A range of complementary data collection techniques was used to capture the richness and complexity of children’s experiences. First, participant observation was conducted throughout the intervention through detailed field notes capturing interaction, drawing behaviour, emotional expression, interpersonal engagement, and responses to activities. Second, all drawings produced during the programme were collected and analysed as visual texts; across the 20 participants, a total of 200 drawings were obtained. Third, semi-structured phenomenological interviews were conducted with each participant. As children shared their drawings, they were invited to explain what they had drawn, the meaning of the symbols and colours used, the feelings represented, and any changes they had noticed over the course of therapy. Interviews were audio-recorded and transcribed with parental consent, and ranged from 20 to 40 min in length; the semi-structured format allowed for consistency across interviews while permitting flexibility to explore individual experiences.

### 3.2 Data analysis procedure

Data analysis followed Moustakas's (1994) transcendental phenomenological procedure, applied across all four data sources—interview transcripts, field notes from participant observation, reflective dialogue records, and the drawings. The analysis proceeded through the following explicit steps:

- *Epoché (bracketing)*: Before formal coding began, the researcher documented personal assumptions and expectations about art therapy and resilience in a reflexive journal, in order to approach the data as openly as possible (see Researcher Role and Reflexivity, above).
- *Horizonalization*: Every statement in the interview transcripts and field notes that was potentially relevant to children’s experiences of drawing was listed and given equal initial value, without judgement of its importance relative to other statements.
- *Reduction to invariant meaning units*: Overlapping, repetitive, or vague statements were removed, and the remaining statements were condensed into invariant meaning units capturing non-repetitive, non-overlapping aspects of participants’ experiences.
- *Clustering into themes*: Meaning units were grouped into thematic clusters through constant comparison across participants and across the four data sources. Drawings were coded for recurring visual motifs and compositional changes over time, and reflective dialogue notes and observation records were used to corroborate, extend, or complicate codes generated from the interviews. A coding matrix tracked how consistently each cluster was supported across sources for each participant.
- *Textural description*: For each theme, a textural description was written summarising what participants experienced, incorporating verbatim quotations from interviews and reflective dialogue.
- *Structural description*: Imaginative variation was used to consider how each experience occurred—the underlying conditions and structures shaping, for example, the shift from fear-based to agency-based drawing.
- *Synthesis*: Textural and structural descriptions for all four themes were integrated into a composite description of the essence of participants’ experience of drawing in art therapy, reported in section 4.5.

The four themes reported in the Results were not specified in advance; they were derived inductively through this process of horizonalization, clustering, and comparison across the interview, observational, reflective-dialogue, and visual data. Where the four data sources converged on a similar experiential pattern, this convergence was treated as stronger support for a theme; discrepancies among sources were retained and discussed in the analysis rather than resolved by privileging one source over another.

Several measures were used to strengthen the trustworthiness of the analysis. Credibility was supported by prolonged engagement, member checking with participants, and consultation with therapists who worked directly with the children. An audit trail documenting methodological decisions throughout the research process supported dependability, while reflexive journaling and independent peer coding checks (described above) supported confirmability. Transferability was supported by providing a detailed description of the research context, participant characteristics, intervention structure, and analytical procedures, so that readers can judge the applicability of the findings to other settings.

Data collection began only after approval from the Ethics Committee of Wenzhou University, Zhejiang, China (Approval No: WZ-0124-3086). Written informed consent was obtained from parents, and children provided assent prior to participation. Confidentiality was maintained through the use of pseudonyms, secure handling of data files, and restricted access to research materials. All participants were informed that participation was voluntary and that they could withdraw at any time without penalty. Parental consent and child assent forms explicitly covered the use and publication of children’s drawings and anonymised quotations from their words in academic outputs; no drawings containing identifying information (such as names or recognisable personal details) were reproduced, and any incidental identifying details in the reproduced images were digitally removed prior to publication (Figure 1).

**Figure 1:**
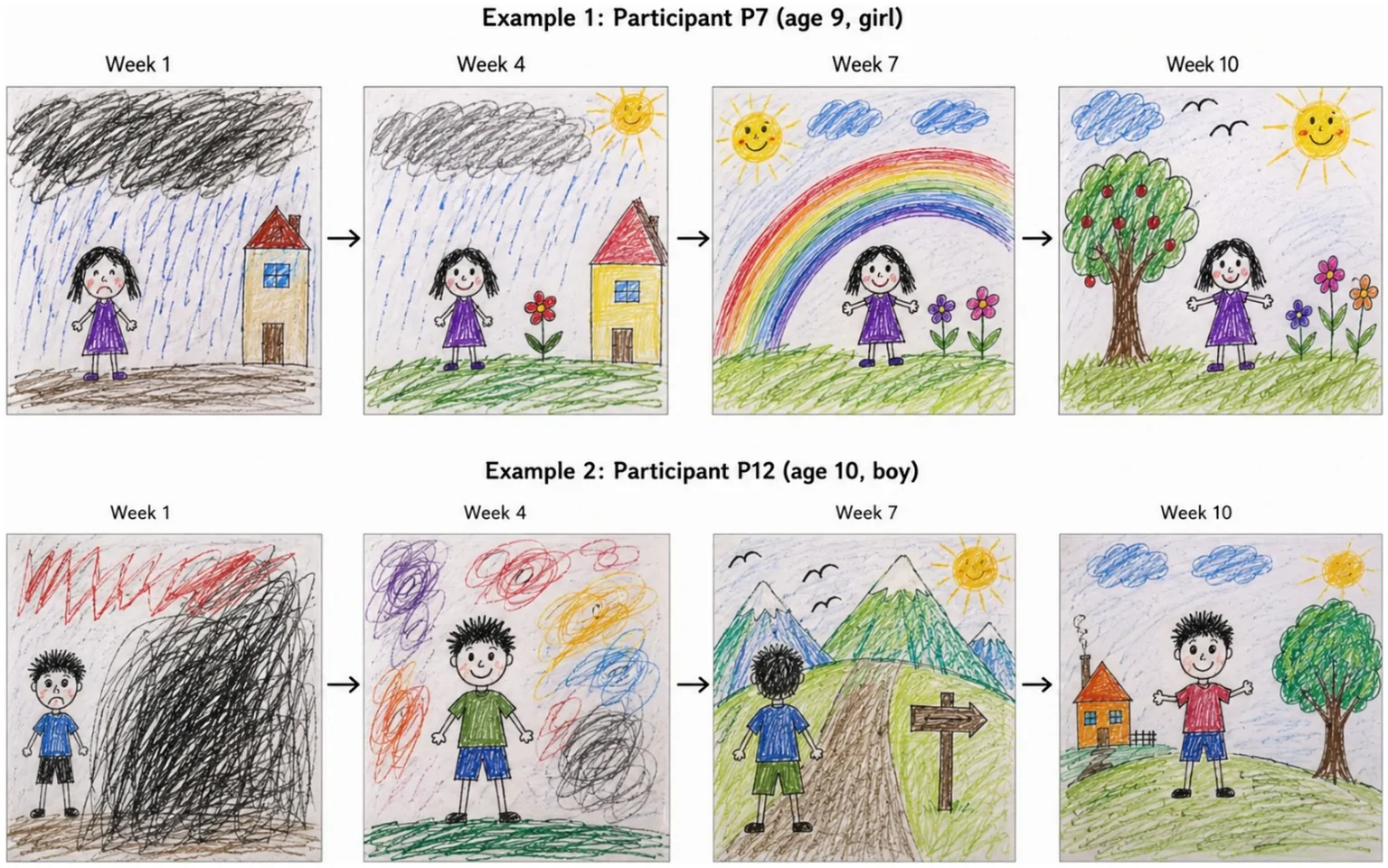
Examples of children’s drawings in different therapy stages.

## 4 Results

The phenomenological analysis yielded four interconnected themes describing children’s experiences of drawing during art therapy and the ways these experiences contributed to emotional resilience. The themes represented an experiential pattern, or perceived progression, within participants’ narratives and drawings, moving from emotional expression to meaning-making, personal transformation, and a more resilient sense of self. Given the qualitative design and the absence of independent pre and post-intervention measures, this pattern is presented as children’s perceived and described experience rather than as evidence of developmental change itself.

### 4.1 Theme 1: drawing as emotional externalisation

The first theme, Drawing as Emotional Externalisation, highlighted children’s experiences of drawing as a safe and accessible space for expressing emotions that were sometimes difficult to verbalise. In interviews and observations, participants described the drawing process as an opportunity to place inner feelings onto paper, making them visible and easier to understand. Many children initially found it difficult to talk about sadness, fear, frustration, and loneliness, but were more willing to engage with these experiences through colour, shape, and visual imagery. Emotional states were often symbolically represented through storms, shadows, isolated figures, and enclosed spaces, and participants described these images as representing experiences they had not previously discussed with others. “When I draw the dark cloud, it is like taking the worry out of my head,” said P2. Similarly, P7 explained, “I don’t always know how to say how I feel to people, but I can show it in my picture.” These accounts suggest that drawing functioned as a way of releasing emotion and psychological distress. Observational data also showed that children became more engaged and expressive as the sessions progressed; initial hesitancy gradually gave way to greater confidence and spontaneity, and artwork-making appeared to ease emotional tension and support self-expression without fear of judgement. P15 reflected, “When I draw, no one says the feeling is wrong.” Participants also noted that drawing allowed them to communicate without needing exact words: children were not required to verbalise emotions in the moment but could depict them pictorially and discuss them afterwards. P9 said, “I started making shapes and then I realised they were my feelings,” a process that appeared to promote emotional awareness and self-recognition. Overall, the results suggest that drawing functioned as an embodied means of communicating emotion, through which children externalised feelings, eased psychological stress, and developed a clearer understanding of their emotional experiences.

All participants exhibited some form of emotional externalisation, although the themes that followed varied in how frequently and how intensely they appeared across cases. Table 3 summarises the number of participants for whom each theme was coded as present, along with corresponding descriptive percentages; these figures are reported for descriptive purposes only and do not reflect statistical significance testing, which was not undertaken given the qualitative design of the study. Emotional externalisation was coded for all 20 participants as the initial experience of the art therapy process. For the majority of participants, symbolic meaning-making emerged as their visual stories became more elaborate. Seventeen of the 20 participants (85%) showed a shift in self-perceived competence, moving from feeling governed by fear toward a stronger sense of agency—a pattern that recurred consistently enough across cases to be considered a notable feature of the dataset. By the end of the intervention, nineteen of the 20 participants (95%) showed indications of emotional resilience and future orientation.

**Table 3:** Distribution of themes across participants.

Theme	Number of participants (*n* = 20)	Percentage (%)	Illustrative indicators
Drawing as emotional externalisation	20	100.0	Expression of hidden emotions, emotional release, use of colours to represent feelings
Symbolic meaning-making through visual narratives	18	90.0	Use of personal symbols, storytelling, metaphorical representations
Transformation of fear into agency	17	85.0	Reduced dominance of fear symbols, emergence of coping characters, active problem-solving
Emergence of emotional resilience and future orientation	19	95.0	Positive self-perception, hopefulness, future-focused imagery, adaptive coping

Percentages reflect the proportion of participants for whom each theme was identified during qualitative coding; they are descriptive counts, not the result of inferential statistical testing.

### 4.2 Theme 2: symbolic meaning-making through visual narratives

The second theme, Symbolic Meaning-Making Through Visual Narratives, highlighted the ways children used artistic symbols and storytelling to make sense of their experiences and construct their own meaning. Drawings became more complex and detailed as therapy progressed, often incorporating motifs such as bridges, trees, mountains, animals, stars, and fantasy characters, each carrying highly personal meanings and emotional associations. For example, P11 explained that a bridge symbolised the transition from fear to confidence: “The bridge means I can go from the scared side to the brave side.” Similarly, P16 linked a growing tree to personal development: “The tree grows because I am learning about myself.” These interpretations illustrate children’s capacity to use visual metaphor to comprehend abstract emotional experience. Interviews suggested that meaning frequently emerged during the act of drawing rather than beforehand, with participants often discovering new understanding while discussing their artwork. P6 remarked, “I didn’t know what the picture was about when I began, but now I think it’s about being okay by yourself.” Emotional understanding was further developed through storytelling around drawings; many participants developed recurring characters representing aspects of themselves, such as fears, hopes, strengths, and challenges. P8 designed a recurring tiger character and explained, “At first, the tiger is hiding, but later he finds that he can try things.” These visual stories gave children opportunities to explore experiences from multiple angles and consider alternative possibilities; challenges were often reframed as a journey or adventure to be met with bravery and perseverance. P14 explained, “It is still a storm, but the person learns how to ride the storm.” Overall, these results suggest that drawing served as an effective means through which children organised their emotional experiences into coherent narratives, supporting self-understanding and the construction of personal meaning.

Meaning-making did not involve eliminating difficult experiences, but reinterpreting children’s relationship with them. Certain visual symbols recurred across the drawings, and while each child’s explanation of a symbol was personal and context-specific, some broad patterns of association were noted across the sample. Table 4 presents these patterns, which are derived primarily from children’s own verbal explanations of their symbols during interviews rather than from predetermined researcher categories; they are offered as illustrative tendencies within this sample rather than as a general symbolic dictionary for interpreting children’s drawings.

**Table 4:** Representative symbols and their phenomenological meanings.

Symbol	Frequency of appearance	Participant interpretation	Associated psychological meaning
Bridge	12	Moving from difficulty to safety	Transition, growth, hope
Tree	14	Growing stronger over time	Personal development, resilience
Mountain	9	Challenge that must be climbed	Perseverance, achievement
River	8	Emotions that keep moving	Adaptation, emotional flow
Sun	15	Better days ahead	Hope, optimism
Animal characters	16	Representation of self	Identity exploration, coping
Storm/cloud	13	Worries or fears	Emotional distress, uncertainty
Path/road	11	Life journey	Future orientation, progress

Associated meanings reflect participants’ own explanations of their symbols during interviews and reflective dialogue and should not be read as fixed or universal interpretations of these visual motifs.

### 4.3 Theme 3: transformation of fear into agency

The third theme, Transformation of Fear into Agency, captured children’s evolving self-image—from a vulnerable recipient of adversity toward a capable agent confronting challenges. Many early drawings depicted fears as large, threatening, and uncontrollable forces, typically represented as monsters, storms, dark shadows, barriers, or dangerous animals; these depictions reflected participants’ experiences of emotional challenges as overwhelming and difficult to manage. P5 explained one of her initial drawings: “The monster is bigger than everything, because that is how the problem feels.” Similarly, P17 said, “I thought there was no way around the dark cloud, so that is why the whole page was dark.” In later sessions, however, marked differences emerged. Fearful symbols remained present but changed in their placement and prominence within the visual narratives: threatening figures decreased in size, distance, or dominance, while supportive characters and coping resources became larger, closer, or more central. P10 noted this shift, saying, “The shadow went away, it didn’t go down with the sun.” Similarly, P1 observed, “Before, she had to wait for someone to save her; now she saves herself.” These statements reflect an emerging recognition that courage does not mean the absence of fear, but the capacity to move through it. Drawing allowed children to engage with threatening experiences in a psychologically safe way and to consider alternative responses; through repeated expression and reflection, participants began to identify strengths and coping resources they had not previously recognised. P13 commented, “The character is still afraid, but she continues to walk.” Observational data corroborated this pattern, showing increased confidence in creative decision-making and greater willingness to discuss challenging situations, consistent with the shift evident in the artwork itself. Together, these findings suggest that art therapy supported the development of agency by helping children symbolically confront their fears, reconstruct their personal narratives, and strengthen their sense of competence and resilience.

### 4.4 Theme 4: emergence of emotional resilience and future orientation

The fourth theme, Emergence of Emotional Resilience and Future Orientation, represented the endpoint of children’s experiences across the art therapy programme. Within their narratives and drawings, participants described and depicted what they perceived as progressively stronger emotional regulation, self-awareness, hope, and adaptive coping over the course of the programme. Initial interviews were frequently characterised by self-doubt, uncertainty, and concern about challenges, whereas later discussions reflected more balanced and positive self-perceptions; children increasingly recognised both their strengths and their challenges and expressed greater confidence in their ability to cope in the future. “Sometimes I still get nervous, but now I know that’s not all I am,” said P3. Similarly, P15 reflected, “Before I only saw parts of myself that were broken; now I see other parts of myself.” Drawings produced in the final sessions frequently depicted paths, open fields, growing trees, bright skies, and future selves, symbolising optimism, growth, and hope for positive outcomes. Participants generally described resilience as being about moving forward rather than the absence of emotional challenge. P20 explained, “There are still times when there are problems, but I think there is a way I can do something about it.” Similarly, P18 said, “My future self isn’t perfect; she keeps trying when she’s in trouble.” Some children also described applying therapeutic strategies outside the art sessions: P12 said, “If I get angry now, I think I sometimes think first and then am angry.” Likewise, P1 shared, “When I feel scared, I think of the bridge picture, and I know that I can cross.” These accounts suggest a degree of transfer of learning to everyday life. Participants also appeared increasingly willing to discuss their feelings and to ask for support when needed, and hope was a recurring element across both drawings and interviews; near the end of the programme, P7 remarked, “I don’t feel stuck anymore.” Taken together, these findings suggest that participants experienced art therapy as contributing to their emotional resilience through increased self-understanding, expanded coping resources, greater agency, and a more positive orientation toward future experiences.

Analysis of the drawings indicated substantial changes over time in how children expressed themselves, in the complexity of their visual narratives, and in how they represented themselves within their drawings; these changes are summarised in Table 5. Across the 200 drawings analysed, a consistent experiential pattern emerged. In early sessions, children tended to use simple images with limited elaboration of feeling states. As the programme progressed, drawings increasingly incorporated brief narratives and more explicit emotional content. By the final sessions, drawings were more organised, reflected greater self-awareness, and depicted a stronger and more coherent sense of self. Together, these patterns suggest that participants’ engagement with drawing was associated with a progression from initial emotional expression toward a more integrated and future-oriented sense of self.

**Table 5:** Changes in drawing characteristics across the art therapy programme.

Drawing characteristic	Early sessions (Weeks 1–3)	Middle sessions (Weeks 4–7)	Final sessions (Weeks 8–10)
Emotional content	Fragmented and implicit	Increasingly explicit	Richly elaborated and reflective
Use of colour	Limited and cautious	More varied and expressive	Diverse and intentional
Narrative complexity	Single objects or scenes	Connected events and characters	Coherent personal narratives
Symbol development	Few symbolic elements	Emerging recurring symbols	Highly personalised symbolic systems
Representation of fear	Dominant and central	Balanced with coping resources	Integrated into broader growth narratives
Self-representation	Passive observer	Active participant	Agentic and resilient protagonist
Future orientation	Rarely present	Occasionally present	Frequently present and optimistic
Overall composition	Isolated images	Greater organisation	Integrated and meaningful visual structures

### 4.5 Composite phenomenological description

The composite phenomenological description synthesises participants’ experiences across all data sources. Although children’s individual circumstances and personal stories varied, a shared experiential structure was evident across the study. For these children, drawing was more than a craft; it was experienced as a relational and transformative process through which emotions could be expressed, explored, and understood. Participants often brought feelings into the therapeutic space that were difficult to put into words. As hands moved across paper, emotions that had previously been ambiguous, concealed, or overwhelming began to take visible form; colours, shapes, symbols, and stories became a language for internal experience. Drawing appeared to create psychological distance from emotional challenges while also allowing for deeper engagement with them: children were not simply representing emotions but actively engaging with, discovering, and reinterpreting them. The artwork allowed fear, sadness, anger, loneliness, and uncertainty to be externalised within a safe, non-judgemental space, and once placed on paper, these feelings became available for reflection rather than remaining purely subjective. Personal meanings developed as participants continued to draw and talk about their drawings, and symbols evolved into stories that, in turn, became ways of comprehending experience. Children drew connections between events, feelings, relationships, and dreams; hardship was not ignored but was woven into broader narratives of learning, adaptation, and growth, supporting an increasingly integrated sense of self and emotional life.

A central feature of this phenomenon was a shift in children’s relationship to fear and vulnerability. Many participants initially found it difficult to face challenges that felt overwhelming and uncontrollable, but gradually began to reposition themselves within their own visual stories. Threatening symbols receded in importance, while symbols of strength, support, and possibility became more prominent, and participants increasingly described themselves not as passive recipients of adversity but as people capable of responding to it. Resilience was experienced as an iterative process of expression, reflection, meaning-making, and action, rather than as the absence of emotional pain—a growing confidence in one’s capacity to face emotional challenges. Children came to understand that they could hold seemingly contradictory states together: feeling both frightened and courageous, hopeful and sad, uncertain yet open to possibility. Emotional difficulty increasingly came to be seen as something to be explored, understood, and worked through, rather than avoided. The final drawings and interviews reflected a strong orientation toward growth, with participants increasingly imagining futures characterised by confidence, connection, kindness, perseverance, and self-acceptance; they described an ability to learn from difficulty and adjust to new situations. Hope, in this account, did not stem from the absence of obstacles but from a conviction that obstacles could be met through creativity, support, and inner strength. In summary, the essence of the phenomenon can be described as follows: children used art therapy to make sense of their embodied emotional experiences, to strengthen their personal agency, and to develop a lived sense of emotional resilience—with drawing serving as a connective thread between feeling, meaning, vulnerability, strength, present challenge, and future possibility (Figure 2).

**Figure 2:**
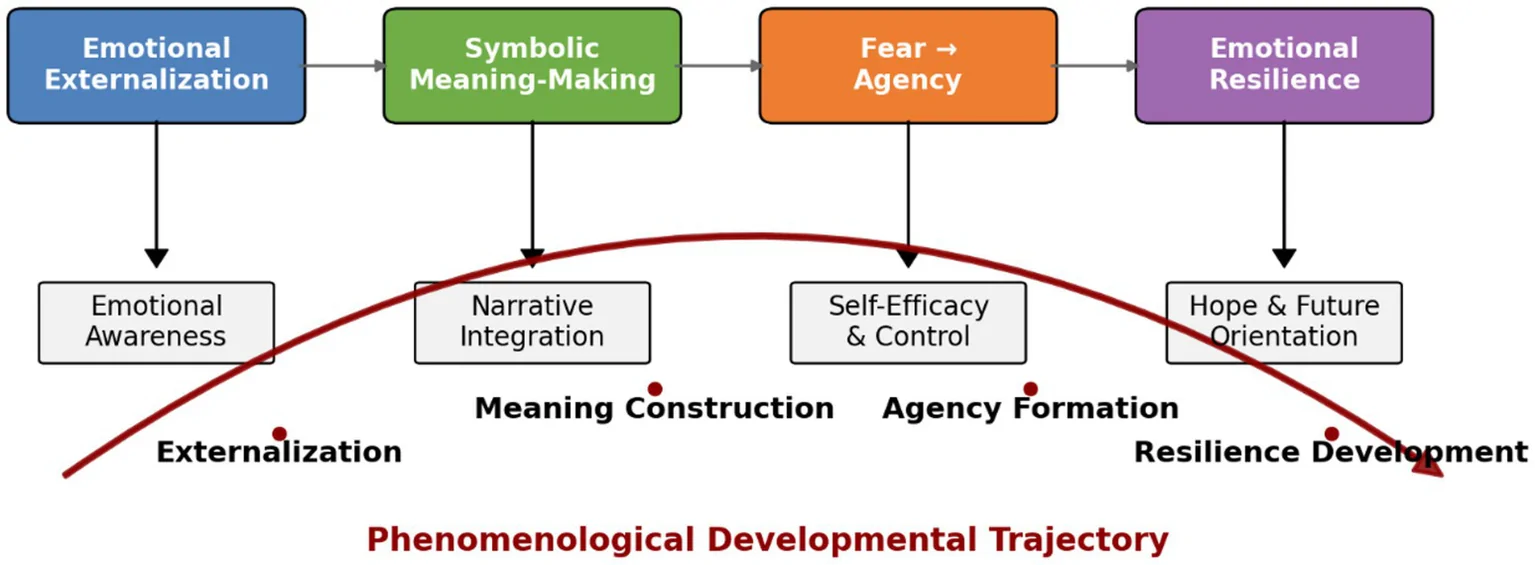
Phenomenological model of children’s emotional resilience development through art therapy.

## 5 Discussion

The findings suggest that art therapy supported children’s emotional experience by enabling them to express their feelings, make sense of those feelings, and develop strength in relation to them. From a phenomenological perspective, participants did not appear to experience drawing merely as an artistic activity; rather, they described it as something lived through, which allowed feelings to become visible and, in turn, more understandable. The data indicate that children used art to communicate emotions in ways that were difficult to achieve verbally. Verbalising feelings is often difficult for children, and some participants described having no other way to express distress in words; drawing offered an alternative that did not depend on direct verbal disclosure. In addition, expressing emotions through drawing appeared to create a degree of psychological distance from distressing experiences, which participants described as making even difficult emotions feel more manageable. These findings are consistent with prior research suggesting that drawing can serve as both an expressive and a regulatory tool for children (Razali et al., 2025; Brechet et al., 2022).

The second theme, symbolic meaning-making through visual narratives, suggests that children were able to interpret and re-imagine their emotional experiences through recurring imagery such as bridges, trees, storms, and animals, which represented adversity and growth and allowed children to build individual, personal symbolic systems. Visual narratives appeared to help children translate abstract emotions into meaningful representations and to view their experiences from multiple perspectives. Symbolic expression offered a new way of engaging with adversity and personal challenge, supporting the construction of coherent narratives around difficult events. Drawing on multiple data sources, children appeared to extend their emotional understanding into a more complex account of their lived experience, developing an enhanced capacity to make sense of, and move forward from, adversity (Sheridan and Van Lith, 2025; Cao and Navarro, 2025).

Themes three and four point to a shift away from vulnerability and toward agency and resilience, with participants increasingly describing themselves as agents able to respond actively to obstacles rather than as passive recipients of adversity. Participants’ images reflected increased confidence, self-awareness, and a stronger sense of personal control over challenging situations, and future-oriented narratives suggested that children increasingly saw themselves as possessing the skills needed to face challenges and adapt to change. Collectively, these findings indicate that engagement in art was associated with positive shifts in self-perception, emotional regulation, and adaptive coping, with the creative process appearing to support communication, self-expression, and psychological flexibility—elements widely considered central to the development of resilience (Sahai and Tiwari, 2026; Mitchell and Meehan, 2022; Cortés and Pérez, 2025; Mastrothanasis et al., 2025a, 2025b).

Overall, the findings suggest that drawing can support a developmental link between emotion and meaning. Children used artistic forms of expression to translate their emotional experiences into symbolic stories, which appeared to support self-understanding, expand coping resources, and foster a more resilient way of relating to difficult emotions. Resilience, in this account, appeared to emerge through the connection between emotional externalisation and the development of symbolic meaning, which in turn supported personal agency. This suggests that art therapy has the potential to be a valuable approach for supporting children’s wellbeing by integrating emotional expression, reflective understanding, and resilience-building within a single process (Sheridan and Van Lith, 2025; Razali et al., 2025; Cortés and Pérez, 2025).

It is important to note that the present study did not include standardised pre and post-intervention measures of resilience, nor independent clinical validation of the observational data by child psychologists. While the phenomenological design was well suited to capturing children’s own meaning-making, the claims made here about developmental or therapeutic change rely on participants’ and the researcher’s interpretations rather than on measurable comparative data. Future research combining this phenomenological approach with standardised resilience measures collected before and after the intervention, and with independent review of observational data by child mental health professionals, would help establish whether the experiential changes described here correspond to measurable improvements in children’s emotional functioning.

### 5.1 Limitations

Several limitations should be considered when interpreting these findings.

- Sample and setting: The study involved a small, purposively sampled group of 20 children across three settings in eastern China. This sample was well suited to an in-depth phenomenological inquiry but limits the generalisability of the findings, which are context-bound and may not transfer directly to children in other cultural, institutional, or age-related contexts.
- Interpretation of visual data: Although the analysis privileged children’s own explanations of their drawings and used reflexive and peer-checking strategies to manage researcher influence, interpretation of visual and symbolic material inevitably involves an element of researcher judgement, and some degree of subjectivity cannot be entirely eliminated.
- Absence of comparison and follow-up data: The study did not include a comparison or control group, nor independent, standardised measurement of resilience before and after the intervention, and no follow-up data were collected after the programme ended. As a result, the study cannot demonstrate causal therapeutic effects or establish whether the changes described by participants were sustained over time.
- Validation of observational data: Observational data were not independently validated by child psychologists or other clinicians external to the research team. As Reviewer 3 rightly notes, collaboration with child development professionals in future studies, alongside measurable pre–post comparative data, would strengthen confidence in the clinical relevance of these findings.
- Scope of claims: Because this is a qualitative, phenomenological study without a comparison group or independent outcome measurement, the findings should be understood as describing participants’ experienced and perceived changes rather than as demonstrating art therapy’s therapeutic effectiveness in a measurable sense.

Ethical approval, parental consent, and child assent were obtained as described in the Methodology, and confidentiality and voluntary withdrawal procedures were followed throughout. As noted above, consent and assent processes explicitly covered the use and publication of children’s drawings and anonymised quotations in this manuscript.

## 6 Conclusion

This research examined children’s experiences of engaging in art therapy and how the act of drawing contributed to the development of emotional resilience among children in China. The study suggests that drawing functioned as more than a creative outlet: it appeared to serve as an important psychological process through which children externalised their emotions, constructed personal meaning, and developed adaptive strategies for responding to challenges. Through symbols, stories, colours, and visual metaphors, children were able to transform complex emotional experiences into forms they could understand and, to some degree, manage. The phenomenological analysis highlighted a progression in participants’ experience—from emotional expression, to the construction of symbolic meaning, to the emergence of agency, and finally to a lived sense of resilience. Over the course of the programme, children increasingly described themselves as capable of engaging with difficulties rather than being overwhelmed by them, with this shift appearing to develop through an interrelated process involving emotional awareness, reflective interpretation, self-understanding, and future-oriented thinking. Art therapy appeared to provide a safe and supportive space in which participants could engage in creative expression while developing emotional regulation, coping strategies, and a more positive sense of self. These findings contribute to the growing body of literature on the therapeutic and developmental significance of art-making for children. As a qualitative, single-group phenomenological study, however, the findings describe children’s experienced and perceived changes rather than demonstrated clinical outcomes; further mixed-methods and longitudinal research, incorporating standardised measures and independent clinical validation, is needed before firm conclusions can be drawn about the long-term therapeutic effectiveness of art-based interventions. With this caveat, the present findings support the value of considering art-based approaches as a complement to educational and psychological support systems aimed at enhancing children’s emotional wellbeing and resilience.

## Data Availability

The original contributions presented in the study are included in the article/supplementary material, further inquiries can be directed to the corresponding author.
